# Pyorrhea Alveolaris: Its Etiology and Treatment

**Published:** 1913-02-15

**Authors:** Thos. B. Hartzell

**Affiliations:** Minneapolis, Minn.


### PYORRHEA ALVEOLARIS: ITS ETIOLOGY AND TREATMENT.

THOS. B. HARTZELL, D.D.S., MINNEAPOLIS, MINN.

Read before the Canadian and Ontario Dental Convention, Hamilton, June 5, 1912.

Pyorrhea Alveolaris, its etiology and treatment, with special reference and emphasis upon the relations which exist between oral sepsis and constitutional infections. For my present purpose I shall use this term to convey the idea of inflammation of the gum margins with progressive destruction of the underlying bone.

Much has been written upon the subject of pyorrhea and after an experience of many years in fighting this enemy of the human race I have reached some practical conclusions which give me the courage to say to you that, though I do not know with absolute certainty whether or not there is a specific micro-organism responsible for this disease, I do know that it is within the power of every dentist to bring about a cure of at least 80 per cent. of all the cases which present themselves for treatment. By a cure I mean that destruction of bone ceases, that the clinical evidences of inflammation pass away. This conclusion is based upon observations and treatment of nearly a thousand cases which it has been the privilege of the writer either to personally treat, or to closely observe and control their treatment by others.

It would give me the keenest pleasure to be able to state authoratively that there is a specific micro-organism responsible for the *continuance* of this so-called pyorrheal inflammation. There is not the slightest doubt in my own mind that when the inflammation is once well established and a lesion of the gum margin exists, thus admitting infection, that the inflammation from that time forward is certainly increased by bacterial influence.

I might ask, what do we actually know regarding the etiology of this disease? I think we can safely say that Dr. E. S. Talbot laid the foundation for the pathology of pyorrhea alveolaris by his research, which he published in book form in 1889, under the title of "Interstitial Gingivitis."

The teachings of Dr. Talbot regarding the etiology of this disease are essentially as follows: That the alveolar process is a transitory structure and particularly subject to inflammatory disturbances. That its blood supply is that of an end-organ, by which he means to point the fact that no great arteries directly feed the tissues contiguous to the teeth, but on the contrary the blood supply comes through the capillary ends which supply these tissues, and is, on that account, more liable to have its blood supply diverted or cut off entirely than are tissues that are supplied by larger arteries. Dr. Talbot also believes that by reason of the evolutionary degeneracy constantly in progress in the human body that the jaws are slowly but surely lessening in size, and the alveolar process thinning in bulk and diminishing in blood supply; consequently the bony process falls a more easy prey to inflammatory disturbances than does the alveolar process of the races which are compelled to use their jaws and teeth more vigorously than the people of the present.

Dr. Talbot also has taught us that the etiological factors of this disease are both constitutional and local, that the constitutional causes are irritating substances absorbed from the bowels as well as retained waste materials due to faulty work by the eliminative organs of the individual. Dr. Talbot also teaches the necessity for eliminating local factors of irritation to the gum margins. He shows conclusively in the illustrations of human and animal jaws which he has prepared, that the inflammatory process eventually involves the deeper portions oi the alveolar bone as well as the margins, but attributes the ultimate changes which occur in the bones to irritants, though he does not definitely state just what these irritants are, he implies that they are of constitutional origin. The bone studies which he made to demonstrate the inflammatory process were, so far as I know, practically the first comprehensive work of the kind undertaken; and, while the deductions which he makes from the studies of these sections are not accepted by all men as correct, the fact remains that the work is extremely valuable even though it has not settled definitely the etiology of this disease.

Many dentist all over the world have recorded their opinions and beliefs regarding this disease; the next contribution worthy of note and careful study, with which the writer is acquainted, is that of Znamensky, of the University of Moscow, in an article on this subject, published in the British Dental Journal for October, 1902. In this article Prof. Znamensky shows a number of well prepared sections of the human jaw demonstrating inflammation of the alveolar process. Prof. Znamensky's conclusions regarding his work were essentially these, that the irritant leading to the bone destruction cause the lesions to begin in the gum margins, extending gradually to the alveolar bone. In this he differs from Dr. Talbot, drawing a somewhat different set of conclusions from the studies of the sections of the tissues undergoing disease which he presents for observation.

A later observer who has done most excellent work is A. Hopewell-Smith, of London, whose observations and studies were presented to the reading public through the columns of the *Dental Cosmos*, under the caption "Pyorrhea Alveolaris: Its Pathohistology," says: "From his personal experiences of cases in which pyorrhea alveolaris was a prominent symptom, the writer is led to the conclusion *that the morbid conditions of the jaws which produce the flowing of pus are not the etiological symptoms of severe metabolic disturbances of the alimentary tract or the vascular system*, but that they are *part and parcel* of them." A strong infection of the oral cavity by means of pathogenic micro-organisms may induce both an extensive pyorrhea in the pockets already deep enough to receive them, in all parts of the mouth, and contemporaneously a pernicious anemia or other lesion which reacts universally on the bodily tissues. Pyorrhea alveolaris does not initiate, but is produced by the same septic cause which leads to general systemic affections, and may, among other diseases, set up pernicious anemia. He sums up the course of events in the following statement: "Atrophy of the bony socket and shrinkage is followed by a widening of the gingival margin and broadening and deepening of the pockets, with hyperplasia of the peridental membrane. If a pathogenic infection occurs, there is a lodgement of pyogenic bacteria in these already suitable pockets, and pyorrhea results, and it may or may not be accompanied by gingivitis and the production of tartar."

He states distinctly that the morbid conditions of the jaws that produce the flowing of pus are not the etiological factors of severe metabolic disturbances of the alimentary tract, but that they are part and parcel of them, which is a way of stating that the disease is constitutional. He also states that a strong infection may induce extensive pyorrhea and also pernicious anemia, and *makes the point* that pyorrhea does not *initiate*, but is *produced* by the same *septic cause* which *leads* to *general systemic infections*.

The work which Dr. Smith has done and the studies he has made of the sections of both healthy and diseased jaws has led him to a different set of conclusions from those of either Znamensky or Talbot, so it would seem that there must be additional evidence gathered before we can agree as to the etiology of this condition.

Dr. Smith concludes his note by stating that at best, "treatment can only be palliative and unfortunately only directed to the prevention of further destruction; and not the *rehabilitation* or *reconstruction* of *parts forever absolutely destroyed*, and supports this statement by showing X-ray pictures which indicate clearly that the teeth there shown have practically lost all alveolar support." These splendid studies still leave us in the dark, and unfortunately leave the impression in the mind of the reader that the treatment of pyorrhea is a comparatively useless and hopeless undertaking.

Talbot has not endeavored to differentiate pyorrheal inflammations into different types, but leaves the impression in the mind of the student that "they are part and parcel," to use the words of Smith, of "severe metabolic disturbances of the alimentary tract and vascular system," and if this were all of the truth it would discourage rather than encourage the average dentist in his effort to combat this disease. Neither of the three authors quoted have pointed out the relation which exists between the tooth's root surface and continued infection. This relationship was pointed out by the author of this paper in an article read before the Northeastern Dental Association and published in the *Dental Cosmos* in 1911, in which the pathology of the root surface was worked out. It was shown that the root surface, when once laid bare through resorptive processes, became a culture bed for many types of bacteria and that the leucocytes failed in their office, because the porous character of the root surface was of such a nature that it was impossible for the leucocytes to reach the bacteria in the pitted surface.

The writer voiced the opinion, which is corroborated by many observers, that a gingivitis invariably preceded a pyorrheal inflammation. That such gingivitis need not be of bacterial origin. Inflammation of the tissues contiguous to the thin edge of the process being entirely sufficient to stimulate resorption of the process margin. The factor which leads to the gingivitis might be of the *simplest character* and *soon* have *passed away*, leaving in its *train nothing* to *mark the fact* that an *irritant* had *ever* been *present*, as far as the *appearance* of the *tissues would show*.

Indeed, the fact that any bone had been lost could scarcely be appreciated except by the keenest search. Nevertheless, real damage has been done from which the patient is never destined to recover unless the pitted root surface thus far exposed to infection be surgically removed. Briefly, the reason for the foregoing statements is found in the porous nature of the root surface itself, and explains why many absolutely healthy individuals show resorption of bony process and the formation of deep pockets; individuals whose eliminative processes are seemingly perfect, the specific gravity of whose urine is normal, whose digestive processes are apparently perfect, but who show marked destruction of the alveolar margins.

The writer fully agrees with the view that destructive inflammations of the alveolar process are accentuated by irritants of constitutional origin; but certainly these constitutional factors do not play a greater or more important part in inflammations involving alveolar process and gums than these same constitutional factors would play in any other local inflammation. A study of the slides presented by Talbot, Smith and Znamensky all show the deepest inflammation nearest the gingivi, and the massing of leucocytes is progressively less as we recede from the gingival margins. This fact seems to have been given little weight by Talbot and Smith in their efforts to determine the point of origin, becoming deeply interested in the grave changes going on in the deeper structures of the advanced case.

The porous cellular nature of the substances of the jaw makes it an easy prey for deep bacterial penetration and reasonably explains the presence of certain foci of inflammation found in the deeper bone when considered in connection with the great injecting power of occlusion. Bacterial invasion begins coincidently with the destruction of the least bit of peridental membrane. The steps being first gingivitis, which may be ever so mild and slight in character leads secondly to destruction of the delicate edge of the process. Such destruction exposes the pitted root surface which predisposes to continued infection. Once infection is admitted then superficial inflammation and subsequent deep-seated inflammation centered along the avenues of least resistance in the bone becomes an accomplished fact. All the studies of bone inflammation presented prove this to be true, and the examination of the deeper structures for bacteria prove them to be present in every case examined by the author, and their presence explains the penetrating character of these inflammations. Why it is that so many observers have ignored, or overlooked, the bacterial holding power of the root surface and the powerful force of occlusion in spreading infection in relation to pyorrheal inflammation is a mystery to the author of this paper. The histological structure of the cementum seems to have entirely escaped the keen analysis which the dental profession gives to the other tissues involved in their sphere of action. A moment's review of the character of the root surface might be of value here. The cementum covering the surface of the root is composed first of a layer of typical bone full of branching bone cells which inhabit its lacunae and canaliculi. As we approach the surface of the cementum it becomes more and more dense, fewer bone cells exist near the surface than in the deeper layers, until finally we reach a very thin, dense layer of cementum in which there are no lacunae and through which very few canaliculi penetrate. This denser layer becomes in fact the foundation upon which nature builds the honey-comb-like surface which gives origin to peridental membrane fibre.

While the profession has not taken the time to analyze the reasons for success or failure of the treatment of pyorrheal inflammation, it is a fact, nevertheless, that Riggs scored success in the treatment of this disease sixty years ago, and that here and there practitioners all over the country have scored successes in the treatment of this disease and have practically proved by their methods of treatment the truth of the statements made regarding the histology of root surfaces, for most successful operators have been surgeons who have advocated and practised the removal of all deposit from root surfaces. Many advocate actually polishing the root surface and in so doing have removed the pitted layer. It is noted that those who have most perfectly attained that perfect condition of root surface which most nearly approximates asepsis show the best results in treatment. Those who have cut too deeply in their effort to remove deposits show recurrent infections.

The massing of the leucocytes is deepest, as was noted in an earlier paragraph, from the gingival margin toward the deeper structures, thinning out and showing less dense masses of leucocytes as we recede from the gingival margins with occasionally dense masses of leucocytes in the deeper structures, due, as I believe, to the presence of bacteria driven by the force of occlusion, which have followed the blood vessels and lodged in the natural cellular openings of the bone. We very seldom find areas of necrotic bone of any considerable size in pyorrhea. What we do find very frequently is carious bone. The bone is lost molecule by molecule rather than dying in masses. This fact is proved clinically in the experience of most operators and it co-incides with the pathological finding of Talbot, Smith and Znamensky. The clinical evidences of bone inflammation in the gums also tend to prove the general statement that these inflammations involve the margins first and the deeper structures later. The thickening of the gum margins, which every observer who has studied these conditions from a clinical standpoint must have noted, indicate as a rule the depth to which the bone is vigorously involved; though, of course, the overlying soft tissues do not always indicate occasional foci of inflammation found in the deeper portions of the alveolar process.

It will be interesting here to call attention to a series of slides presented in an article by Mr. J. F. Collier, in the London *Lancet*, May 7, 1910, page 1261, showing the "progressive destruction of the teeth sockets." Mr. Collier presents first a series of photographs of human and animal jaws which show markedly the progressive destruction of the bone from the alveolar margin toward the root apices and in one slide there is a distinct evidence of condensing osteitis over the region of a cuspid co-incident in the same mouth with rarefying osteitis. Mr. Collier also presents a series of slides showing cellulitis of the gum margins and radiographs of the teeth hidden by these same tumid gums. Mr. Collier's clinical description of his cases are classics and betoken the keen and analytical observer and will well repay anyone who will take the trouble to peruse them.

Condensing osteitis is seldom described but it is a more or less constant feature of pyorrheal inflammation, having been noted by different observers and is beautifully shown in the case of a human skull in the possession of Dr. Wm. Bebb. I express the opinion that could we but find a method of so stimulating bone cells and periosteum after the surgical treatment of root surfaces has been accomplished, so that condensing osteitis would supervene, the problem of saving for years of usefulness many teeth now lost would be solved.

Statements regarding the rebuilding of bone I am well aware are heretical; particularly is this the belief of Dr. Smith, whose earlier statement you will recall was "that the treatment was at best palliative and only directed to the prevention of further destruction and not the rehabilitation or reconstruction of parts absolutely and forever destroyed and not admitting of adoption of certain plastic measures which are used at times in connection with other bones of the body." I am willing to admit that if the plastic measures referred to by Dr. Smith mean bone-grafting, thus far he is probably right, but that new bone is not built around the roots of teeth that have once undergone destructive inflammation is not an impossible thing, but on the contrary may become the rule and not the exception.

It is the experience of the author that teeth previously swinging in their sockets become rigid and that this degree of rigidity progressively increases. If these same teeth be held under a stiff splint for a sufficient period of time I note on the removal of such splint that teeth which one year before were exceedingly loose have become solid. It was recently my privilege to observe a case in the practice of my friend Dr. Logan, who will address you on that phase of the subject relating to diagnosis. In this case a lower central had rotated in the arch and moved forward and was exceedingly loose. Dr. Logan gave this tooth and the teeth adjoining it correct surgical treatment and moved the tooth by orthodontia appliances a considerable distance in the arch, again aligning it with its fellows and completing the operation by placing upon the tooth a proper retaining splint which he allowed to remain the period of a year. He removed the splint in the presence of the writer, April 27, 1912, when it was found that this tooth, which had previously been very loose, standing in a funnel-shaped depression in the bone, had become quite rigid and the tooth, to the casual observer, certainly did not suggest that it had ever been in the loose, unstable condition which cast and picture certainly prove it to have been before the treatment was undertaken.

The form of retaining splints and splint bridges in use by the author have duplicated this experience herein detailed many times. And a series of radiographs made before and after treatment, presented by Dr. Fletcher, of Cincinnati, all bear cumulative evidence that new bone is under certain conditions deposited about the roots of teeth which have lost previously existing alveolar process.

Pyorrhea alveolaris begins in the gum margins. Perhaps no more conclusive argument can be adduced on this point than the fact which every dentist has noticed, that the removal of infected teeth results 99 times out of 100 in cessation of all inflammatory symptoms. The second corroborative fact is, that removal of the infected porous root surface usually checks all types of interstitial gingivitis precipitately, if the work be accurately done. In the observation of 1,000 cases the author has found no exception to this general rule, except in cases of acute diffuse nephritis, diabetes mellitus and certain types of drug poisoning. Barring these conditions, no matter whether the case be one in which the marginal inflammation was induced by an accumulation of salivary calculus above the gum margin which causes the loss of not only alveolar process, but overlying soft tissues as well, or the impaction of food and its decomposition between the teeth, or badly fitted crowns mal-occlusion, acute febrile diseases of childhood, or rough, ragged enamel margins or any other mechanical, traumatic or chemical stimulant locally applied. The result is the same, namely: cessation of inflammatory symptoms and a return to normal color of overlying soft tissues providing the root is "skinned." If, on the contrary, any considerable number of the various types of gingival inflammation and osteitis found in this locality resists this treatment, one would reasonably be justified in concluding that constitutional causes were largely responsible for the presence of such inflammations and the opsonic index has been greatly reduced by the constant absorption of bacterial leucomaines. When one commonly and constantly sees marked and brilliant results follow simple surgical treatment of the root surface in a constantly increasing list of recorded cases one is absolutely compelled to give such statistics credence.

Talbot shows conclusively (page 62, Interstitial Gingivitis), "that calcic deposits on the roots of teeth (below the gum margins) are a result of inflammation and pus infection, and not the cause." Calcic deposits from saliva above the gum margins, on the contrary, the author believes to be a common cause, if not the most common cause, of inflammation of the gum margin. It is quite immaterial to me whether you designate the condition arising from calcic deposits above the gum margin as "salivary calcific pericemental alveolitis," according to Prof. Logan, or whether you call it pyorrhea alveolaris or interstitial gingivitis or Riggs' disease, the net result is the same, namely, loss of bone, loss of teeth, and the initiating factor is a local irritant of some sort or other.

The continuing factor which perpetuates the inflammation while you sleep is mixed bacterial infection. Right here I wish my readers would take notice for there has been many discussions on this point, and if the thought has not occurred to you before, now that this fact is brought to mind, its force should become apparent. Of course, in the saving of teeth an inflammation induced by salivary deposits with a consequent sinking of gum and peridental membrane and bone may not show a marked pus infection and is certainly less difficult to treat than an inflammation which has drilled the alveolar process full of holes, and become richly infected, but just the same the patient will eventually lose his teeth unless this condition is corrected and the deposit of calculus checked, and lest I forget it I wish to state that since listening to Dr. Black's recent contribution to our literature showing that over-feeding and hypernutrition is the cause of salivary deposits, the author has experienced the satisfaction of noting the cessation of salivary calculus deposits in the mouths of five patients who were induced to markedly decrease their daily food supply and with the added benefit of increased vigor as a direct result of such curtailment of food, which resulted in more perfect assimilation of the lessened portion.

### THE PART WHICH BACTERIAL INFECTION PLAYS IN BONE INFLAMMATION.

The great amount of work which has been done in an effort to discover specific micro-organisms for various diseases has gradually brought to light certain basal facts in regard to micro-organisms, which it would be well to note in discussing the relationship which they bear to pyorrhea. First, it has been found that certain micro-organisms resist growth on artificial media for considerable periods of time. Some organisms refuse to grow on artificial media entirely, others refusing to grow on artificial media when separated from certain other micro-organisms usually found growing with them. It is a rare thing indeed to find pure cultures of any form of bacterial growth.

Dr. A. F. Schafer, of Bakersfield, Cal., is of the belief that all infections are "mixed infections," and that, except in rare instances, there is no such thing as an infection by a single species of micro-organism. While one species may predominate, the pathogenic process engendered by it is accelerated and intensified by the complicating presence of other organisms of other species, in other words, in the course of an infectious disease the symptoms are due not only to the effects of a single species of organism (the specific infection), but to the influence of other organisms whose pathologic role is not insignificant, but which must be reckoned with in any successful scheme of therapeutics.

Dr. Schafer further believes that the human subject is at all times the host of a great variety of organisms and harbors these pathogenic bacteria without harm to himself during periods of physiological resistance at par, and in the absence of any solution of tissue continuity. When the resistance is below par, or a solution of continuity of tissue occurs, the bacteria harbored by the human host assume pathological significance.

Furthermore, he contends that certain diseases, as typhoid fever, pneumonia, tuberculosis, rheumatism, and others, are symptomatic manifestations of the preponderance in the patient of the toxic and destructive products of the peculiar species of organism to which the etiology of the disease is usually ascribed, as bacillus typhosus in typhoid fever, bacillus tuberculosis in tuberculosis, etc. In addition, the symptoms are due in part at least to the destructive action of certain materials produced by complicating organisms which are always present in great variety and number.

It is now a commonly accepted idea that in pulmonary tuberculosis the great danger to the patient, the difficulty of the treatment, and many of the most notable symptoms, such as loss of weight, high temperature, disturbance of circulation, purulent expectoration, destruction of tissue, etc., *are due to the complicating organisms*, and if the so-called mixed infection can be checked or eliminated, efforts may be directed against the bacillus tuberculosis with far greater success than has heretofore been possible in the treatment of this condition.

The administration of bacterial vaccines to patients suffering from infection not infrequently fails of effect because the truth of the above assumption is not recognized, especially when the treatment, being based upon the opsonic theory, consists in the use of a vaccine made from a single species of organism isolated from the patient. Bacterial vaccines made from a single species of organism prove successful in some cases, but the multiplicity of "combined" bacterial vaccines now in use points to the rapidly developing conclusion that the great majority of patients require something more than treatment with a vaccine made from one organism; the success attending the use of bacterial vaccines made from a number of different species, even when used in cases apparently due to one species, points to the liklihood of this theory being correct.

The fact that certain pathogenic micro-organisms can be harbored in the tissues for considerable periods of time without harm to the body is due to two facts, the first and perhaps most important one is, that the blood serum has a certain normal resistance, an overcoming power which is inimical to their growth. This material has been called an opsonin. Wright and Douglas, of London, demonstrated in 1903 "that the blood serum contained substances which acted upon the bacteria, making them attractive food for the white blood corpuscles, and this substance they called 'opsonin,' from the Latin *opsonare*, meaning to cater, or prepare food for;" and Wright developed a technic by which he could determine the amount of opsonins present in the blood serum of any human being as compared with any other human being. This anti-body in blood serum is the most vital characteristic in animal resistance to bacterial invasion. If the creative power of the individual for the development of opsonins is minus then the individual becomes an easy prey to infection. The second fact is the physical need for particular forms of food demanded by the micro-organisms which, if not found in the tissue, obliges such micro-organism to exist quiescent or harmless. Perhaps the particular element lacking for the rapid growth of such organisms depends upon the presence of another organism, which, being planted in conjunction with the first, provides the necessary element for the growth of the second, and it then becomes virile and active, producing characteristic symptoms which are referred to it by observers without noting the fact that it is dependent for its pathogenicity upon what may be under ordinary circumstances an absolutely harmless organism. It seems likely that bacterial growths which we find in pyorrhea pockets depend vitally on the presence of two or more micro-organisms. These are not new ideas, but they are sometimes lost sight of by those enthusiasts who are in search of a specific micro-organism.

Dr. Timothy O'Leary of Tufts College, of Boston, in an article published in the *Dental Cosmos*, January, 1910, voices the opinion "that the fusiform bacillus so constantly found in pyorrheal pus is to be looked upon as the essential infective agent," and we all know it has resisted growth in pure culture, and thus far I have found it absolutely impossible to make a vaccine of this organism, or even to obtain it in a sufficiently pure state to estimate opsonic index of the individual to it in whose tissues it has been plentifully found. It is not unlikely therefore, that the fusiform bacillus may be, as O'Leary believes, the specific organism of pyorrhea, though Goadby found that in many of his cases the opsonic index was usually low to staphylococcus and the pneumococcus, and this was also the experience of Madalia, who reports 32 cases treated by vaccines made from these organisms with marked benefit.

TREATMENT.

Prof. Znamensky, describing his method of treatment, based upon the pathological conclusions from his studies, which were, in brief, that the disease was one which commenced in the gum margins and progressed toward the bone, adopted a method of treatment which consisted in scraping out the bone around the necks of the teeth. At the time his article was written he had 742 cases with 2,415 scraped-out sockets which had been affected with purulent discharge, and says that the scraping out of the affected bone of the socket and the gingival sac is the only rational means for radical and rapid treatment, and he adopted local anesthesia to make it possible to do this operation painlessly, and owing to the painlessness of the operation he had a better opportunity to remove the tartar so cleanly that afterwards it was unnecessary to inject acids to dissolve out any of the remaining particles of tartar.

I wish you to note here that Znamensky, in explaining his great success in the treatment of his cases by curetage of the alveolar margins makes it plain that he deals very carefully with the root surfaces, and I believe that it is probable that the greatest benefit of his treatment is derived from the perfection with which he removes the dead putrescent surface of the root exposed in the pockets at the same time and by the same method that he accomplished the removal of the tartar.

I bring this point out because in the treatment of my own cases, while scrupulously removing any carious bone found in the bottoms of the pockets I have found it unnecessary in the great majority of cases to scrape or curette the alveolar margins and find that the accurate skinning of the root surface induces rapid healing and cessation of pus flow without the sacrifice of additional bone.

The methods of Znamensky, while successful, require the copious use of local anesthetics and necessitate the removal of much bone upon which the continued usefulness of the tooth absolutely depends. Znamensky dwells not at all on the bacterial holding power of the root surface, and while believing the disease in a great majority of cases to be local in character, gives comparatively little heed to the penetrating power of the bacteria themselves, nor does he mention power of mastication to drive the bacteria in the pockets into the vascular channel.

A careful student of pathology in general must inevitably arrive at the conclusion that the great majority of pathological lesions are due to the introduction of living bacterial organisms into the tissues. The great diseases with which we are becoming more and more familiar are found to be dependent upon inoculation. Bacteriological researches tend to create in our minds the belief that all the important diseases depend upon direct inoculation into the tissues of living organisms which are either specific or mixed infections. Therefore, the first great logical conclusion regarding this disease should be to *shut out infection.* First, for the specific reasons that we wish to save our teeth, and second, because any door of entry held constantly open for the introduction of living organisms into the circulation must be an enormous menace to the life of the individual. If we wish to plant tetanus we must have a door of entry, a break in the shin or mucous membrane. If we wish to plant tuberculosis, yellow fever, diphtheria, rheumatism, etc., a point of inoculation must be provided. The penetrating character of all these types of bacteria, causative of these various diseases, is well known, and the greatest danger is a break in the skin or mucous membrane. We devote untiring vigilance to the exclusion of organisms from accidental wounds, and our greatest surgeons give their closest attention and best thought to this matter. We ventilate our buildings with washed and strained air. We inveigh against spitting in the streets to prevent the lungs from picking up bacterial infections. Lungs guarded by tracheal mucous membrane armed with mucous glands to wash off, and cilia to whip out micro-organisms. We carefully scrutinize our food, for fear we may pick up harmful organisms in that food which may gain an entry through some trifling break in the mucous membrane of the alimentary tract, and at the same time overlook, to a great extent at least, the largest door of entry for the inoculation of harmful infections—a door of entry cunningly hidden from the eye—and for that reason doubly dangerous, infected root surfaces.

The root surface when once infected never ceases to be a culture bed until the tooth is finally extracted, or until that root surface is robbed of its bacterial holding power. While for a considerable period of time the leucocytes of the body can and do engulf and assimilate millions of bacteria and call forth opsonins in opposition to the harmful bacterial leucomaines; the leucocytes in time tire and the creation of opsonins after a time grows fainter. Goadby aptly states this fact, "that small but considerable dosage with bacteria and their products from a local focus tends to gradually break down immunity". (Page 109, *London Practitioner*, Jan., 1912.) He states further in the same article, "that of many mouth infections an exceedingly small local cause may produce suddenly generalized and widespread infection without apparent exacerbation of the mouth disease preceding such infection."

In stating this truth regarding infections, Goadby is discussing arthritis deformans in relation to mouth infections. If the genral surgeon knew that in most of the cases upon which he operated there existed in direct contact with bleeding vessels a dead surface loaded with a bacteria, varying in extent from a quarter of an inch to five square inches he would be horrified and would probably recommend the wholesale extraction of teeth. But such is actually the case. Great prominence has been given the crypts of the tonsil as culture beds and points of inoculation for bacterial infection, and indeed the tonsil is frequently a point of entry for many types of infection. Alfred Mantel, first pointed out the relationship of the tonsil as a point of entry to rheumatoid infection in 1887.

Dr. Frank Billings, of Chicago, in the *Illinois Medical Journal* for March 1912, cites ten cases of multiple arthritis which were produced by streptococcic infection of tonsils, pure cultures of streptococcus were obtained in these cases, which became free of joint disturbances upon enucleation of the tonsils. He also reports a case of arthritis deformans due to pyorrhea, and it is undoubtedly true that the tonsil is frequently a point of inoculation for streptococci. He also reports subacute and chronic parenchymatous nephritis due to the same streptococcus which he finds in the tonsil, and the great majority of the medical profession seem to believe that the tonsil is a place to admit systemic infections and advise tonsilectomy and practice it with great success, but nevertheless few individuals suffer tonsil infections as compared to the vast number of individuals who suffer the same character of infections from pyorrhea pockets. In the first place the tonsil is normally lined with columnar epithelium. The pyorrhea pocket is not so protected, but, on the contrary, contains a dirty root surface heavily infected, constantly in contact with bleeding and broken blood vessels. Hence, much more likely to introduce micro-organisms into the general circulation than is the tonsil. It is more likely to do this because the tooth itself must act as a hypodermic syringe plunger. With every movement of occlusion, the teeth, bearing, as they do, according to Prof. Black, hundreds of pounds per day of pressure, must, on occlusion, drive out into the circulation with each closure of the jaws the bacteria which multiply in the pyorrhea pockets, forcing these bacteria to scatter through the body of the jaw and enter the lymphatics, as well as gaining direct access to the blood stream. This explains the foci of inflammation found scattered through the body of the bone in the studies of Talbot, Smith and Znamensky, and it also explains the great prevalence of tubercular glands in children. The writer of this article has three cases of septic endocarditis upon record traced directly to infection by way of pyorrhea pockets.

I also wish to report a typical case of pyorrhea alveolaris, patient aged 50, male. Molar and bicuspids in both lower and upper arches being freely movable in sockets, having lost the bone to about one-half of the original depth of the sockets, pus discharging freely from the sockets about the teeth, temperature normal, specific gravity of urine 1018, no albumen, no sugar. Patient reports a tender area in the stomach wall. Diagnosis, ulcer of the stomach accompanied by chronic dyspepsia. Patient has had treatment for ulcer of the stomach for two years with but temporary benefit. The treatment in this case was first extraction of two of the loose teeth, followed by accurate planing of the root surfaces of all the teeth which had lost alveolar process. Absolute cessation of pus-flow. Gums resume normal tint, and after two months no tenderness in the region of the ulcer; digestion about normal.

Case 2. Male, aged 48, chronic pain and tenderness in the masseter muscles of left side. Tenderness of the sublingual glands and tortocollis. Tenderness of the left shoulder joint. Examination of the mouth revealed dead pulp in left lower 8 with free pus discharge from deep pyorrhea pockets. Chronic acid indigestion with constant eruction of gas after the ingestion of food. Treatment, extraction of loose left lower 8. Planing of the root surface of all the teeth affected by pyorrhea. Pockets were pencilled with tincture of iodine. After two weeks rheumatic pains in shoulder and tenderness of sublingual glands disappeared. Digestion improved. End of the fourth week, all inflammatory symptoms contiguous to the teeth absent. Teeth no longer tender on occlusion. Patient has resumed vigorous mastication of food. End of two months, all symptoms of dyspepsia absent. This case No. 2 is typical of a group of five cases in which joint involvements have been present from one to three years which have all disappeared upon the stamping out of oral infections. In this connection I draw your attention to the report of three cases of severe mouth infection accompanied by arthritis deformans reported by Goadby in the *London Practitioner* for January, 1912, which cases were clearly traceable to mouth infections and yielded to local treatment, reinforced by vaccines made from organisms isolated from the gum infections.

Goadby was able to produce well-marked joint inflammations in animals by inoculating them with living organisms obtained from the mouths of these patients. I draw your attention to these cases to accentuate the fact that bacterial infections can and do occur through the medium of pyorrhea pockets that such infections may involve any tissue in the body dependant upon the character of the infection. The bacteria found in the human mouth and alimentary tract depend upon habitat, occupation, foods and internal resistance of the individual. In the matter of foods, Metchnikof has proved conclusively that those individuals who live largely upon buttermilk or clabbered milk replace the putrefactic bacteria in the bowel with the lactic acid bacillus, clearly to the benefit of the host, and all will admit that the individual who habitually cleanses carefully his teeth and oral surfaces will have fewer and less varied forms of bacterial growth than the individual with the habitual dirty mouth. The vast importance of oral infections to the general welfare of the body was never better illustrated than by that series of cases reported by Dr. Henry S. Upson, Professor of Neurology in the Western Reserve Medical School, in the *Dental Cosmos* for May, 1910, in which he shows conclusively nine cases of insanity due to painless dental disease. Of these nine cases operated upon dentally, six have recovered, two are improved, and one remains unimproved. The accumulation of well authenticated records of this sort certainly must increase the importance of dental disease in its relation to the welfare of the human body. Not only do they find dental lesions operating to bring about the loss of the teeth themselves, but we find that these lesions open a door to infections which may cause pernicious anemia, septicemia, rheumatism, neurasthenia, severe forms of dyspepsia, and acute insanity, tuberculosis, actinomycosis hominous, and nephritis." There is not the slightest doubt in the mind of the author that pneumonia is a mouth-planted disease. The absolute necessity for a point of inoculation for all germ infection and the constant presence of lesions under the gum margins, thus providing a door of entry for these infections, should re-awaken the interest of every dentist and physician to the necessity for preventing such lesions and a sense of responsibility to see that such lesions, once incurred, are speedily healed. This is easily possible. The recognition on the part of the dentist of the necessity for surgical interference in all cases of alveolar inflammation should rest on one specific fact, namely that wherever alveolar process has been lost, exposing broken vessel ends and pitted root surface to infection, treatment is indicated, and that treatment should consist of first, the elimination of every possible form of local irritation, scrupulous cleanliness of the necks of the teeth maintained by daily care on the part of the individual, and the planing of the root surface wherever fibre-bearing surface has been exposed. The removal by the curette or bur of any dead bone recognized in the alveolar process and the free use of iodine pencilled into the pockets to destroy as many as possible of the bacteria which could not be removed surgically. These measures should be reinforced by careful attention to diet and the avenues of elimination. Flush the blood stream by generous drinking of water. This will increase the activity of the skin and alimentary tract and help to maintain that nicely-adjusted balance between waste and repair.—September, 1912, *Dominion Dental Journal.*

